# Differences in chunking behavior between young and older adults diminish with extended practice

**DOI:** 10.1007/s00426-017-0963-6

**Published:** 2017-12-21

**Authors:** J. S. Barnhoorn, E. H. F. Van Asseldonk, W. B. Verwey

**Affiliations:** 1 Cognitive Psychology and Ergonomics, MIRA, University of Twente, Enschede, The Netherlands; 2 Department of Biomechanical Engineering, MIRA, University of Twente, Enschede, The Netherlands; 3 Human Performance Laboratories, Department of Health and Kinesiology, Texas A&M University, College Station, TX USA

## Abstract

Previous research found reduced motor chunking behavior in older adults compared to young adults. However, it remains unclear whether older adults are unable to use a chunking strategy or whether they are just slower in developing them. Our goal was to investigate the effect of extended practice on the development of chunking behavior in healthy older adults. A group of young and a group of healthy older adults between 74 and 85 years of age visited the lab on 2 days. A sequence of 3 and a sequence of 6 elements were both practiced 432 times in a discrete sequence production task. We found that age differences in chunking behavior, as measured by the difference between initiation and execution of the sequence, diminish with extended practice. Furthermore, in older, but not in young adults, slow responses that are often interpreted as the first response of a next motor chunk were associated with a finger that was also slow during performance of the random sequences. This finding calls for more attention to biomechanical factors in future theory about aging and sequence learning.

## Introduction

Older adults show impaired performance in the physical and cognitive domains. These impairments are associated with increased difficulty in developing new motor skills (e.g., Wu & Hallett, 2005). Since Western societies are aging rapidly, it is important to better understand age-related changes in cognition and motor performance. Age-related cognitive decline has been reported over a broad range of cognitive abilities (Harada, Natelson Love, & Triebel, 2013), including processing speed (Harada et al., 2013; Salthouse, 2004), and working memory (Borella, Carretti, & De Beni, 2008; Verhaeghen & Salthouse, 1997). Cognitive changes are normally accompanied by ongoing physical decline and these factors together explain limitations in motor performance and learning. For example, reduced hand function in older adults is explained simultaneously by deterioration at the biomechanical level (e.g., joints, muscles, bones) and changes in neural control (Carmeli, Patish, & Coleman, 2003; Seidler et al., 2010). However, more research is needed on how changes in motor learning can be explained by changes in cognitive and physical capabilities.

Sequence learning is one of the major paradigms that have been used to study motor learning. A recent framework for understanding the learning and production of sequences is the dual processor model (DPM, Abrahamse, Ruitenberg, de Kleine, & Verwey, 2013). The DPM is based on the results from many studies using the discrete sequence production (DSP) task but also incorporates features of other sequence learning models (e.g., Hikosaka, Nakamura, Sakai, & Nakahara, 2002; Keele, Ivry, Mayr, Hazeltine, & Heuer, 2003). The architecture proposed in the DPM consists of a cognitive processor that is dependent on attentional resources and a motor processor that does not require attentional resources. Together, these processors enable three modes of sequence production (Ruitenberg, Verwey, Schutter, & Abrahamse, 2014; Verwey & Abrahamse, 2012). In the reaction mode, the cognitive processor sequentially translates each target into an appropriate response, which is then carried out by the motor processor. As sequence learning progresses, performance may improve because associations between elements begin to develop, in which case the learner has progressed into the associative mode. Finally, in the chunking mode, series of successive elements in a sequence are integrated into a single representation that can be loaded into a motor buffer as a whole after being triggered by the cognitive processor. This single representation is not necessarily a motor representation, but may also be central-symbolic (Verwey, Shea, & Wright, 2015). Response time patterns in the chunking mode are characterized by multiple features. The first key press is typically slower than later elements. This delay is caused in part by time uncertainty and in part by the loading of a representation consisting of multiple upcoming elements into the motor buffer. After the first response, subsequent elements are quickly performed in one sweep. The DPM predicts that “the difference between the first (initiation) and later (execution) response times (RTs) increases considerably with practice because of the increasing possibility to prepare the later key presses” (Abrahamse et al., 2013). Hence, an increase in the difference between initiation and execution suggests increased chunking. Longer sequences are often divided into multiple motor chunks and one or more slower elements in the RT pattern are taken to indicate that a next chunk is initiated (Abrahamse et al., 2013). While the term chunking was originally based on this division process, research has shown that, in accordance with the DPM, chunking behavior is reflected in multiple aspects of sequence learning and performance (Acuna et al., 2014).

Previous research on motor chunking in older adults has focused on multiple aspects of chunking behavior. For example, in a study by Verwey (2010), most older participants (aged 75–88 years old) were found to remain reliant on external stimuli and the difference between initiation and execution key presses did not increase like it did in the young participants. Similar results were later found for a group of middle-aged participants (Verwey, Abrahamse, Ruitenberg, Jiménez, & De Kleine, 2011). Bo, Borza and Seidler, (2009) found that a lower proportion of the older adults in their study, compared to the young participants, divided a long 12-element sequence into multiple motor chunks. This study also showed that when the older participants did chunk, their chunks consisted of fewer elements than those of young adults. The movements performed in most studies on chunking consisted of keyboard presses. However, reduced chunking abilities in older adults have also been shown in a task with forearm flexion–extension movements (Panzer, Gruetzmacher, Fries, Krueger, & Shea, 2011; Shea, Park, Wilde, & Braden, 2006). Clearly, previous research has provided important insights into age-related differences in chunking but since all these studies used a relatively limited amount of practice, an important question remains: Are older adults unable to develop chunking strategies or do they simply need more practice to develop them?

### The current study

Our main goal was to investigate the effect of extended practice on the development of chunking behavior in healthy older adults. We used a DSP task with a 3- and a 6-element sequence. These sequence lengths are similar as used in previous studies on chunking in older adults (Verwey, 2010; Verwey et al., 2011). We provided 3 times as much practice compared to these previous studies as each of the 2 sequences was repeated 432 times, spread over 2 consecutive days. This number of practice trials is quite typical for DSP studies with young participants (Abrahamse et al., 2013).

Our aim was to see how older adults perform in the light of DPM’s predictions. We hypothesized that they would require more extensive practice than young adults to increase chunking behavior. This chunking behavior would be indicated by an increasing difference between the first and following key presses of each sequence (i.e., the Initiation–Execution Difference or IED). Although it was unknown how much practice would be needed, we expected differences in chunking behavior between older and young adults to gradually reduce during the second day of practice, and perhaps even disappear. In previous research, slow elements in the sequence were taken to indicate the start of a new motor chunk, also called a concatenation point (e.g., Ruitenberg et al., 2014). However, especially with the older adults it may well be that slow elements are due to increased biomechanical variability (e.g., Contreras-Vidal, Teulings, & Stelmach, 1998). To investigate this potential problem, we here determined for both age groups whether slow elements in a learned sequence correspond with slow fingers, as identified when performing random sequences, in older and young adults.

In addition to these primary goals, we measured visuospatial working memory capacity, explicit sequence knowledge, and processing speed in order to enhance our understanding of the factors contributing to differences in sequence learning between older and young adults. Visuospatial working memory (VSWM) capacity has been shown to be associated with the length of motor chunks developed by older and young adults (Bo et al., 2009), and we were interested whether this would be the case for the IED too. Because previous research showed that explicit sequence knowledge is correlated with the initiation–execution difference (Verwey, 2010), we also measured explicit sequence knowledge. Finally, processing speed has not previously been related to chunking behavior, but since processing speed has been suggested to play a central role in many age-related cognitive differences (Salthouse, 1996), we here explored its relationship with chunking behavior and sequence execution, too.

## Methods

### Participants

The young participants were students participating for course credit. Older adults, in the range of 74–85 years old, were recruited via local media. The older applicants were invited for participation only when they reported that none of the following applied: severe motor problems including use of a wheelchair or limitations in using the fingers or arms; history of neurological problems; arthritis or rheumatism; or color blindness. Of the older adults who initially visited the lab, 7 were excluded and replaced due to: pain in the wrist (1); limitations in using the fingers (1); more than 30% errors during the last 4 blocks of day 1 (4, participation ended after visit 1 for these participants); and more than 80% errors during the random sequence test phase (1). The 18 older adults (age = 79 ± 3.5, 13 females) that were eventually included for analysis had a score on the Montreal Cognitive Assessment (MoCA) of 26 ± 2.5 on a scale of 0–30. None scored below 22, the threshold for mild cognitive impairment (Freitas, Simões, Alves, & Santana, 2013). Of the 18 young participants tested (age 21 ± 1.2, 7 females), no participants were excluded or replaced. All participants were right-handed as confirmed by the Edinburgh handedness inventory (Oldfield, 1971). The ethics committee of the University of Twente, Faculty of Behavioral, Management and Social Sciences, approved the study and all participants provided informed consent.

### Procedure

**Fig. 1:**
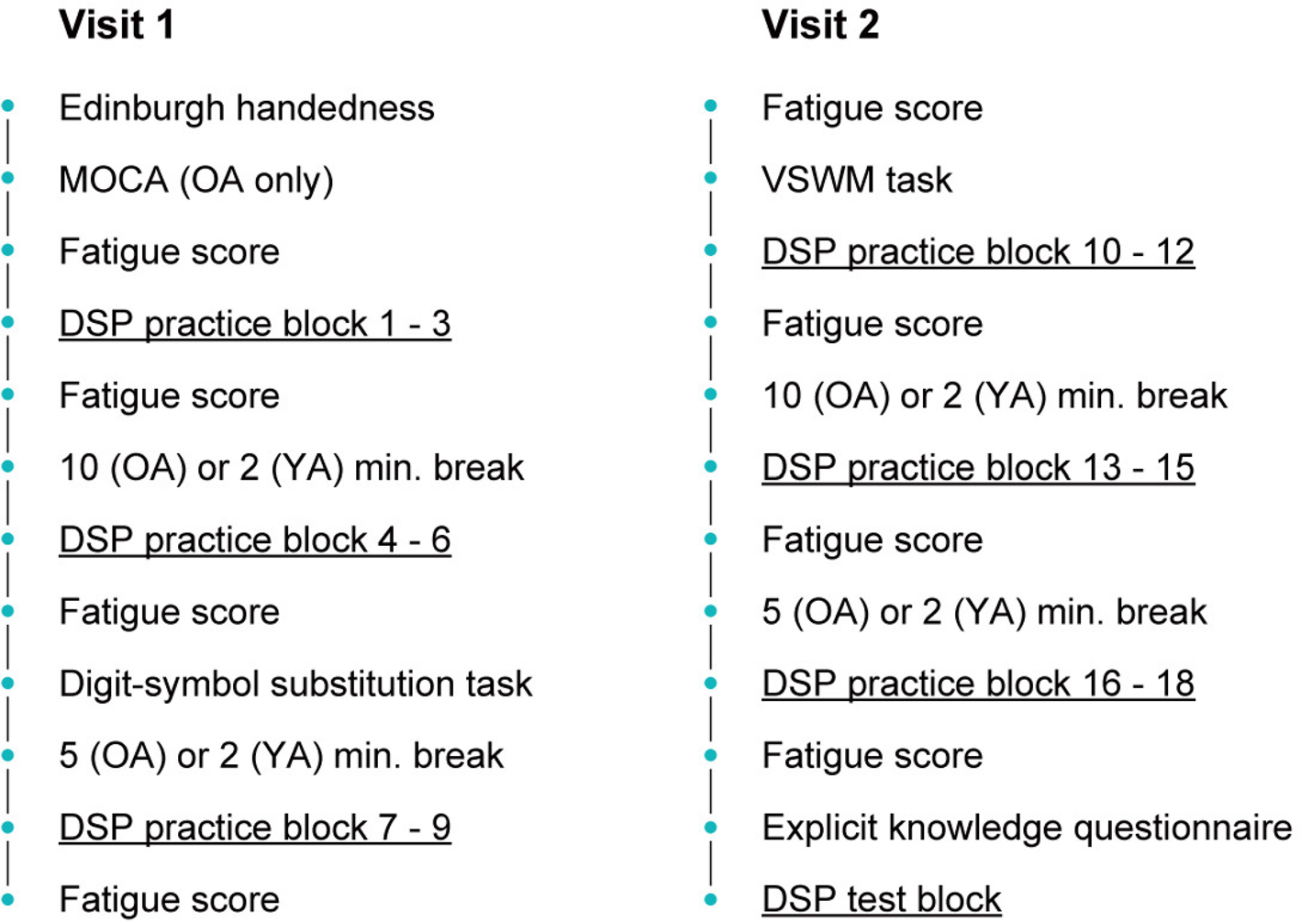
Study procedure. The main activity participants performed was DSP practice, the other tasks and questionnaires were interleaved with the DSP blocks. Note that the older adults (OA) were provided with longer breaks than the young (YA) participants

All participants visited the lab on two consecutive days. The older participants received an information letter and the Edinburgh handedness form (Oldfield, 1971) at home prior to participation. For them, the MoCA was administered at the start of the visit (Nasreddine et al., 2005). Then, on both days, participants completed a series of DSP blocks with breaks and additional questionnaires interleaved (see Fig. 1). After every three DSP blocks, an ad-hoc self-report fatigue scale (11 point Likert scale) was administered. After block six on the first day, a 90 s digit-symbol substitution test was administered to measure processing speed (Wechsler, 1955). A visual array comparison test to measure visuospatial working memory capacity was included at the start of day two. In this task, participants view a sample array of 2–8 colored squares for 100 ms, then, 900 ms later, a test array is presented and the participant is asked whether the test array is identical to the sample array (see Barnhoorn, Döhring, Van Asseldonk, & Verwey, 2016). After the last DSP practice block on the second day, a questionnaire measuring explicit sequence knowledge was administered. In this awareness questionnaire, participants were first asked to write down the order of the elements using the letters corresponding to the keys they had used (while the keyboard remained in sight). Then, the target locations were shown on the screen again and participants were asked to point out the sequences by pointing with their index finger. Finally, participants were asked to select their sequences from 2 lists of 12 sequences, 1 for each sequence length. At the end of the first visit, older participants were offered a stress ball to relax hands and fingers.

### Discrete sequence production task

Participants practiced one 3-element sequence and one 6-element sequence in random order. Across participants, the keys of the sequences were rotated over sequential positions (e.g., G, L, D becomes J, D, F) so that over participants, all fingers were used equally often at each sequence position. Furthermore, the sequences did not involve repeated elements, and did not involve regularities like trills and runs. The DSP task was presented on a 22″ wide-screen monitor using E-Prime 2.0. On the display, black outlines of six 28 × 28 mm placeholders were aligned horizontally with 14 mm spacing in between them. The background was white. These squares corresponded to the buttons D, F, G, J, K and L on the regular QWERTY keyboard on which the participants rested their ring, middle and index fingers of both hands. The spacing between the third and fourth placeholder was 56 mm, a letter H was presented here. A square was filled green when it became the active target, after which the participant pressed the spatially compatible key. Each time the correct key had been pressed the next stimulus was displayed. This continued until the sequence was completed. Note that the response-stimulus interval was zero and, hence, the RT is equal to the inter-key interval. We denote the first response RT1, the second RT2, etc. After an incorrect key had been pressed the message “Wrong” was displayed for 1500 ms. When no key was pressed for 20 s, the error message “No response” was displayed for 1500 ms. In both cases, a new trial started. Before each trial, the empty placeholders were displayed for 1000 ms. Pressing any key during this period resulted in a 1500 ms error message “Too early” after which the trial was repeated. After each trial, a 1250 ms white screen was presented.

Participants were instructed that they would learn two fixed sequences of key presses during practice. The task consisted of 18 practice blocks with 48 trials each, rendering 864 practice trials in total. Each block comprised 2 sub-blocks, between which a 40-s break was provided. Between two full DSP blocks, participants were given a 2-min break during which the error percentage and mean RT in ms were displayed on the screen, older participants enjoyed a longer break after every three DSP blocks (see Fig. 1). The test phase consisted of two sub-blocks. One sub-block involved the familiar sequences; the other sub-block involved random sequences that were generated online (without immediate stimulus repetitions). The order of these test phase sub-blocks was counterbalanced over participants.

### Analyses

For all RT analyses, we excluded the first trial (i.e., sequence) of every sub-block and trials containing an error. Of the resulting dataset, we excluded 1.47% of trials with a mean trial RT that was above a threshold of the mean trial RT plus 2.5 × standard deviation of mean trial RTs in that sub-block. When Mauchly’s test indicated that assumptions of sphericity were violated we applied the Greenhouse–Geisser correction, and reported corrected p values and original degrees of freedom. The proportions per block of trials (sequences) with an error were arcsine transformed before analysis (Winer, Brown, & Michels, 1991). We report explicit knowledge based on the sum of the number of elements correctly written down and the number of elements correctly pointed out during the explicit knowledge questionnaire (correct elements were counted from the start to the first mistake; maximum explicit knowledge score 18).

## Results

### Practice phase general results

**Fig. 2:**
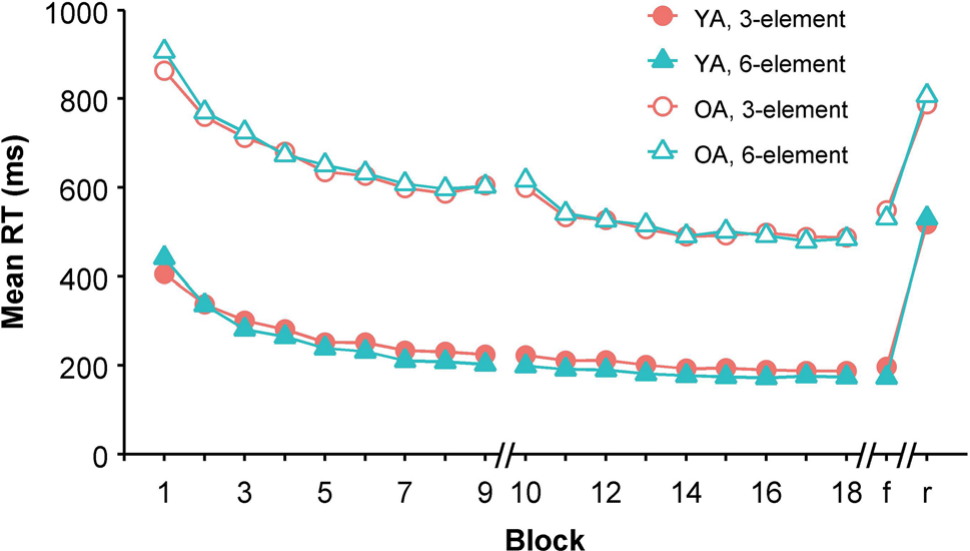
RT development during the practice and test blocks (*r* random, *f* familiar), the order of the test phase sub-blocks was counterbalanced, Day 1 ended after Block 9

Response times of the 3-element sequence were submitted to a mixed 2 (Age group) × 18 (Block) × 3 (Serial position) ANOVA with Age group as the between-subjects variable. Mauchly’s test indicated that the assumption of sphericity was violated for Block, *χ*^2^(152) = 664.8, *p* < 0.001, *ε* = 0.226, Serial position, *χ*^2^(2) = 24.7, *p* < 0.001, *ε* = 0.655, and their interaction, *χ*^2^(594) = 1938.3, *p* < 0.001, *ε* = 0.136. The older adults were substantially slower than their young counterparts (594 vs. 239 ms), *F*(1, 34) = 53.5, *p* < 0.001, *η*_p_^2^ = 0.61 (see Fig. 2). Furthermore, performance improved over Blocks, *F*(17, 578) = 83.1, *p* < 0.001, *η*_p_^2^ = 0.71 and differentially so for older and young adults as indicated by a Block × Age group interaction, *F*(17, 578) = 7.6, *p* < 0.001, *η*_p_^2^ = 0.18. Serial position showed a main effect, *F*(2, 68) = 90.1, *p* < 0.001, *η*_p_^2^ = 0.73, which is in line with the first key press being slower than subsequent key presses. Serial position interacted with Age group, *F*(2, 68) = 6.3, *p* = 0.010, *η*_p_^2^ = 0.16. A significant Serial position × Block interaction, *F*(34, 1156) = 9.7, *p* < 0.001, *η*_p_^2^ = 0.22, provides a first indication that the key presses developed differently over time.

Response times of the 6-element sequences were submitted to a mixed 2 (Age group) × 18 (Block) × 6 (Serial position) ANOVA with Age group as the between-subjects variable. The assumption of sphericity was violated for Block, *χ*^2^(152) = 765.0, *p* < 0.001, *ε* = 0.184, Serial position, *χ*^2^(14) = 61.2, *p* < 0.001, *ε* = 0.605, and could not be computed for their interaction. Here too, older participants were much slower than young (601 vs. 224 ms), *F*(1, 34) = 50.6, *p* < .001, *η*_p_^2^ = 0.60. Furthermore, performance improved over Blocks, *F*(17, 578) = 80.8, *p* < 0.001, *η*_p_^2^ = 0.70, and there was a Block by Age group interaction, *F*(17, 578) = 6.3, *p* < 0.001, *η*_p_^2^ = 0.16. Serial position showed a main effect, *F*(5, 170) = 32.6, *p* < 0.001, *η*_p_^2^ = 0.49, which is in line with a relatively slow first key press. Serial position interacted with Age group, *F*(5, 170) = 4.1, *p* = 0.009, *η*_p_^2^ = 0.11. A significant Serial position × Block interaction, *F*(85, 2890) = 6.9, *p* < 0.001, *η*_p_^2 ^= 0.17, indicates again that the key presses developed differently over time.

Arcsine transformed error proportions for the 3-element and the 6-element sequence were submitted to mixed 2 (Age group) × 18 (Block) ANOVAs with Age group as between-subjects variable. The assumption of sphericity was violated for Block for the 3-element sequence, *χ*^2^(152) = 204.2, *p* = 0.005, *ε* = 0.608, but not for the 6-element sequence. For the 3-element sequence as well as for the 6-element sequence error proportions, none of the main or interaction effects reached statistical significance. This suggests that the speed differences over time and between age groups were not due to speed-accuracy effects. During practice, older participants made errors on 6% of the sequences, young participants on 5% of the sequences.

Development of fatigue was tested separately for both days with mixed 2 (Age group) × 4 (Time of measurement) ANOVAs on the 11-point fatigue scale with Age group as between-subjects variable. The assumption of sphericity was violated for both day one, *χ*^2^(5) = 28.6, *p* < 0.001, *ε* = 0.729, and day two, *χ*^2^(5) = 61.6, *p* < 0.001, *ε* = 0.471. The main reason this analysis was performed was to see if practice had differential effects of fatigue development in young and older adults. Critically, the Time by Age interaction was not significant for either day 1, *F*(3, 102) = 2.0, *p* = 0.133 or day 2, *F*(3, 102) = 1.6, *p* = 0.215. This shows that older and young participants did not experience significantly different amounts of increased fatigue and, thus, that differences in fatigue development did not affect differences in learning. The main effect of Age group showed that young participants scored higher on the fatigue measurement on day 1, *F*(1, 34) = 9.8, *p* = 0.004, *η*_p_^2^ = 0.22, and day 2, *F*(1, 34) = 5.3, *p* = 0.027, *η*_p_^2^ = 0.14. We think that this effect is indicative of a general strategic difference in responding baseline between the age groups that is irrelevant for the current study. The effect of Time on fatigue score was significant on both day 1, *F*(3, 102) = 17.3, *p* < 0.001, *η*_p_^2^ = 0.34, and day 2, *F*(3, 102) = 7.4, *p* = 0.004, *η*_p_^2^ = 0.18, indicating that all participants experienced increasing fatigue during the experiment.

### Test phase general results

**Fig. 3:**
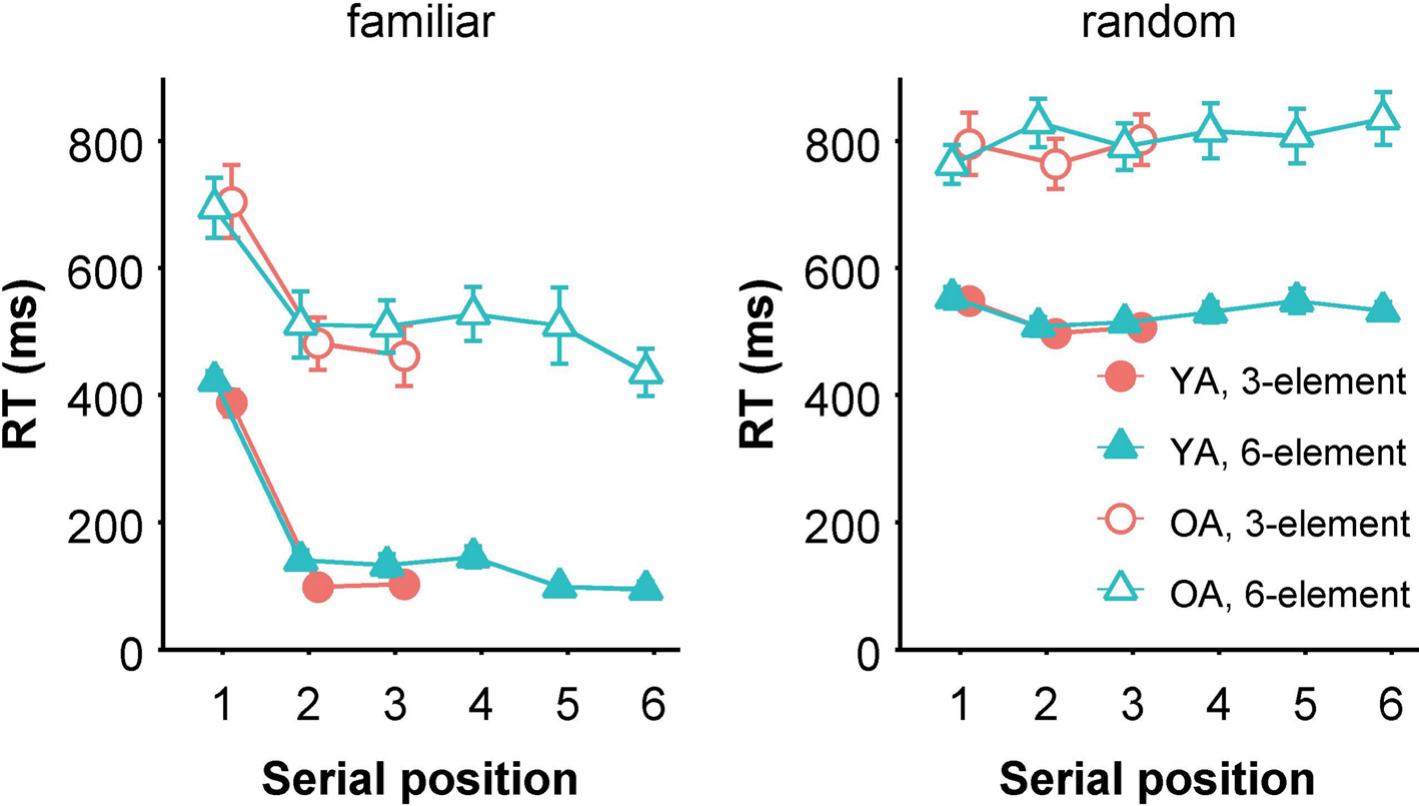
RT per serial position during the test phase. Error bars denote standard error. Note that the left plot seems to suggest that the 6-element sequence was performed as one large chunk, since no clear slow elements are visible. This is not the case, this RT profile simply results from averaging over multiple participants who have slow elements at different locations (Verwey & Eikelboom, 2003)

A mixed 2 (Age group) × 2 (Familiarity: Familiar vs. Random) × 3 (Serial position) ANOVA was conducted on 3-element sequence RTs with Age group as between-subjects variable. The assumption of sphericity was violated for Serial position, *χ*^2^(2) = 21.7, *p* < 0.001, *ε* = 0.675, and for the Serial position by Familiarity interaction, *χ*^2^(2) = 6.4, *p* = 0.041, *ε* = 0.850. A main effect of Age group confirmed the age-related slowing (668 vs. 357 ms), *F*(1, 34) = 61.0, *p* < 0.001, *η*_p_^2^ = 0.64. In general, participants were slower in the Random than in the Familiar condition (652 vs. 373 ms.), *F*(1, 34) = 340.2, *p* < 0.001, *η*_p_^2^ = 0.91, confirming that participants had gained sequence knowledge during practice. This effect was stronger for the young than for the older participants, *F*(1, 34) = 7.5, *p* = 0.010, *η*_p_^2^ = 0.18. Serial position showed a main effect, *F*(2, 68) = 46.4, *p* < 0.001, *η*_p_^2^ = 0.58. An interaction between Familiarity and Serial position, *F*(2, 68) = 90.9, *p* < 0.001, *η*_p_^2^ = 0.73, supports the notion that in general, motor chunking was used in the Familiar but not in the Random sequences (see Fig. 3).

A mixed 2 (Age group) × 2 (Familiarity: Familiar vs. Random) × 6 (Serial position) ANOVA on RT was conducted for the 6-element sequence data as well. The assumption of sphericity was violated for Serial position, *χ*^2^(14) = 27.9, *p* = 0.015, *ε* = 0.723, and for the Serial position by Familiarity interaction, *χ*^2^(14) = 36.5, *p* = .001, *ε* = 0.717. The effect of Age group was confirmed (669 vs. 352 ms), *F*(1, 34) = 76.7, *p* < 0.001, *η*_p_^2^ = 0.69. The effect of Familiarity was significant too (669 vs. 352 ms), *F*(1, 34) = 482.8, *p* < 0.001, *η*_p_^2^ = 0.93. Similar to the 3-element sequence results, the advantage of the Familiar over the Random sequences was larger for young than for older participants, *F*(1, 34) = 8.2, *p* = 0.007, *η*_p_^2^ = 0.20. The main effect of Serial position was significant, *F*(5, 170) = 25.5, *p* < 0.001, *η*_p_^2^ = 0.43, and differed between Age groups, *F*(5, 170) = 3.9, *p* = 0.007, *η*_p_^2^ = 0.10. Again, like with the 3-element sequences, Familiarity and Serial position showed an interaction suggesting a different RT pattern in the Familiar than in the Random condition, *F*(5, 170) = 35.9, *p* < 0.001, *η*_p_^2^ = 0.51.

Arcsine transformed error proportions during the test phase for the 3 and 6-element sequences were submitted to 2 (Age group) × 2 (Familiarity: Familiar vs. Random) ANOVAs. Participants made more errors in the Random (old 10%, young 11%) than in the Familiar (old 5%, young 6%) condition in the 6-element sequence, *F*(1, 34) = 9.5, *p* = 0.004, *η*_p_^2^ = 0.22, and in the 3-element sequence, *F*(1, 34) = 6.8, *p* = 0.013, *η*_p_^2^ = 0.17. For both the 3 and 6-element sequences, the main effects of Age group and the interactions were not significant.

### Initiation–execution difference

For the 6-element sequences, we defined three types of responses: *initialization* consists of RT1, the initiation of the sequence; a *slow element*^1^ is any response after RT1 that is consistently slower than its neighboring responses; all other RTs are *execution* responses. We categorized responses on a block-by-block basis for all blocks with fixed sequences (18 practice phase blocks and one test phase sub-block). A response was classified as a slow element when two one-tailed, paired samples *t* tests with alpha set to 0.1 indicated it to be slower than its neighboring responses in the current block. Consequently, a sequence could have 0, 1 or 2 slow elements. This approach is in line with previous research (Bo et al., 2009; Ruitenberg et al., 2014). Note that RT2 and the last RT can never be a slow element when using this method. This is partly because they do not have two suitable neighbors to compare with^2^ and partly because, in line with the aforementioned studies, it is assumed that RT2 is part of the first chunk and the last RT is part of the last chunk. Accordingly, we defined all 3-element sequences to consist of one initialization followed by two execution responses. For the practice phase data, we defined an initiation–execution difference (IED) to gauge the amount of chunking, as the difference between RT1 and the mean of the execution responses, also on a block-by-block basis. The reason we did not include slow elements in the IED calculation (e.g., averaged with RT1) is that these may be slower because of biomechanical factors, especially in older adults, while our aim was to assess a cognitive process that would be associated with the IED.

**Fig. 4:**
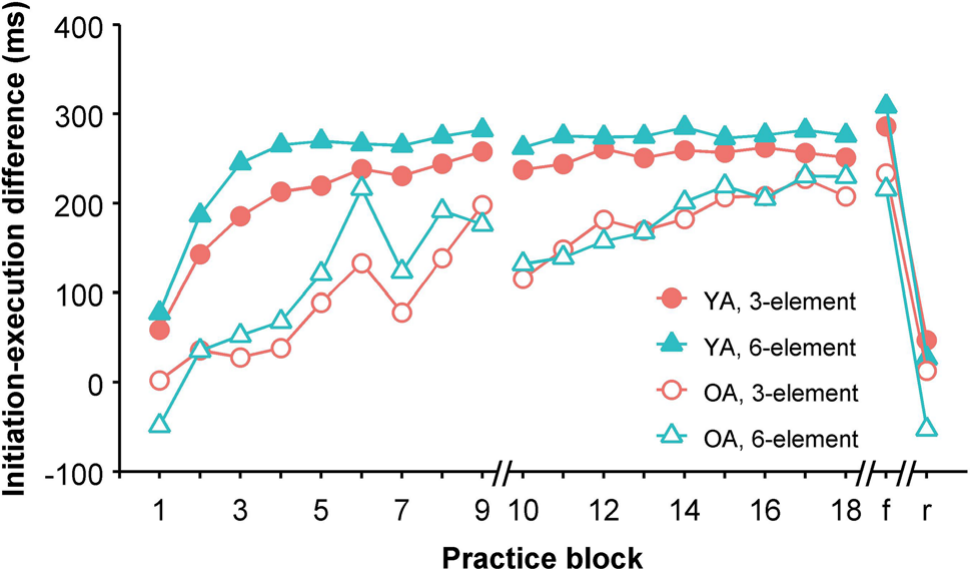
IED development during the practice and test phase

To test whether the older participants benefited from the additional practice provided on the second day, we submitted the IED for each practice block of the second day to mixed 9 (Block) × 2 (Age group) ANOVAs for each sequence length. The assumption of sphericity was violated for the 3-element sequence, *χ*^2^(35) = 190.6, *p* < 0.001, *ε* = 0.313, as well as for the 6-element sequence, *χ*^2^(35) = 122.2, *p* < 0.001, *ε* = 0.367. For the 3-element sequence, the main effect of Block was significant, *F*(8, 272) = 3.9, *p* = 0.017, *η*_p_^2^ = 0.10; the main effects of Age group and the interaction did not reach significance. For the 6-element sequence, the main effect of Block was significant too, *F*(8, 272) = 5.2, *p* = 0.003, *η*_p_^2^ = 0.13, and the main effect of Age group did not reach significance either. This time, however, Block interacted with Age group, *F*(8, 272) = 3.5, *p* = 0.019, *η*_p_^2^ = 0.09, suggesting that the older participants benefited more from the additional practice than the young participants did (see Fig. 4). However, the differential development during day two may be due to a larger decrease of performance at the start of day two. To test this, we submitted the IED’s from the last blocks of each day to a mixed 2 (Age group) × 2 (Block: block 9 vs. 18) ANOVA. The main effects of Block and Age group did not reach significance, but the Block by Age group interaction again showed that older participants benefited more from the second day of practice than the young, *F*(1, 34) = 4.8, *p* = 0.036, *η*_p_^2^ = 0.12. Additional one-tailed t tests confirmed that the older participants showed a higher IED at the end of day two than at the end of day one for the 6-element sequence, *t*(17) = 2.2, *p* = 0.02 (one-tailed), while the young participants did not, *t*(17) = 1.6, *p* = 0.93.

For the test phase, IED was submitted to a 2 (Familiarity: Familiar vs. Random) × 2 (Age group) mixed ANOVA. For the 3-element sequence, IED was higher in the Familiar than in the Random condition, *F*(1, 34) = 127.5, *p* < 0.001, *η*_p_^2^ = 79. The main effect of Age group and the interaction were not significant. For the 6-element sequence, IED was higher in the Familiar condition as well, *F*(1, 34) = 112.7, *p* < 0.001, *η*_p_^2^ = 0.77. This time, the main effect of Age group did reach significance, *F*(1, 34) = 9.3, *p* = 0.004, *η*_p_^2^ = 0.22, the Familiarity × Age interaction was not significant.

### Slow elements and biomechanical variation

To establish whether there is an age-related difference in variance between fingers, we calculated the SD of the median finger RTs per participant using the random sequence test block. As expected, older participants showed a larger SD (72.0 ms) than their young counterparts (38.8 ms), *t*(34) = 3.9, *p* < 0.001 which is consistent with the presence of larger biomechanical variation among fingers in older adults.

To investigate whether the occurrence of slow elements in older adults is associated with biomechanical factors like a stiff finger^3^, we used the data from the random sequence test block (excluding RT1), where sequence knowledge is irrelevant, to determine the median RT of each finger. Finger speed was then calculated per finger as the difference between a finger’s median RT and the mean of the median RTs of all the fingers used. A positive value for finger speed indicates a finger slower than average, a negative value a finger faster than average. Because of the normalization, the average finger speed is zero.

Using the aforementioned t test method, we found for all participants slow elements for at least one of the practice blocks. To determine whether these slow elements often occur at sequential positions that are performed with a ‘slow finger’ for a participant, we counted the number of times each finger was used at a slow sequence element. Then, we took the average finger speed of these fingers, taking into account in how many blocks these fingers were associated with slow elements. For example, a participant might display no slow elements in the first 6 blocks of the practice phase, a slow element at sequential position 3 from block 7–8, and at position 4 from block 9–18. Say that the left index finger was used at sequential position 3, and the right middle finger at position 4, with a finger speed of, respectively, 40 and 70 ms. For this participant, the mean finger speed at the slow elements is than [(2 × 40) + (10 × 70)]/12 = 65 ms, indicating that the fingers used at slow elements were also slow in the random sequence blocks.

**Fig. 5:**
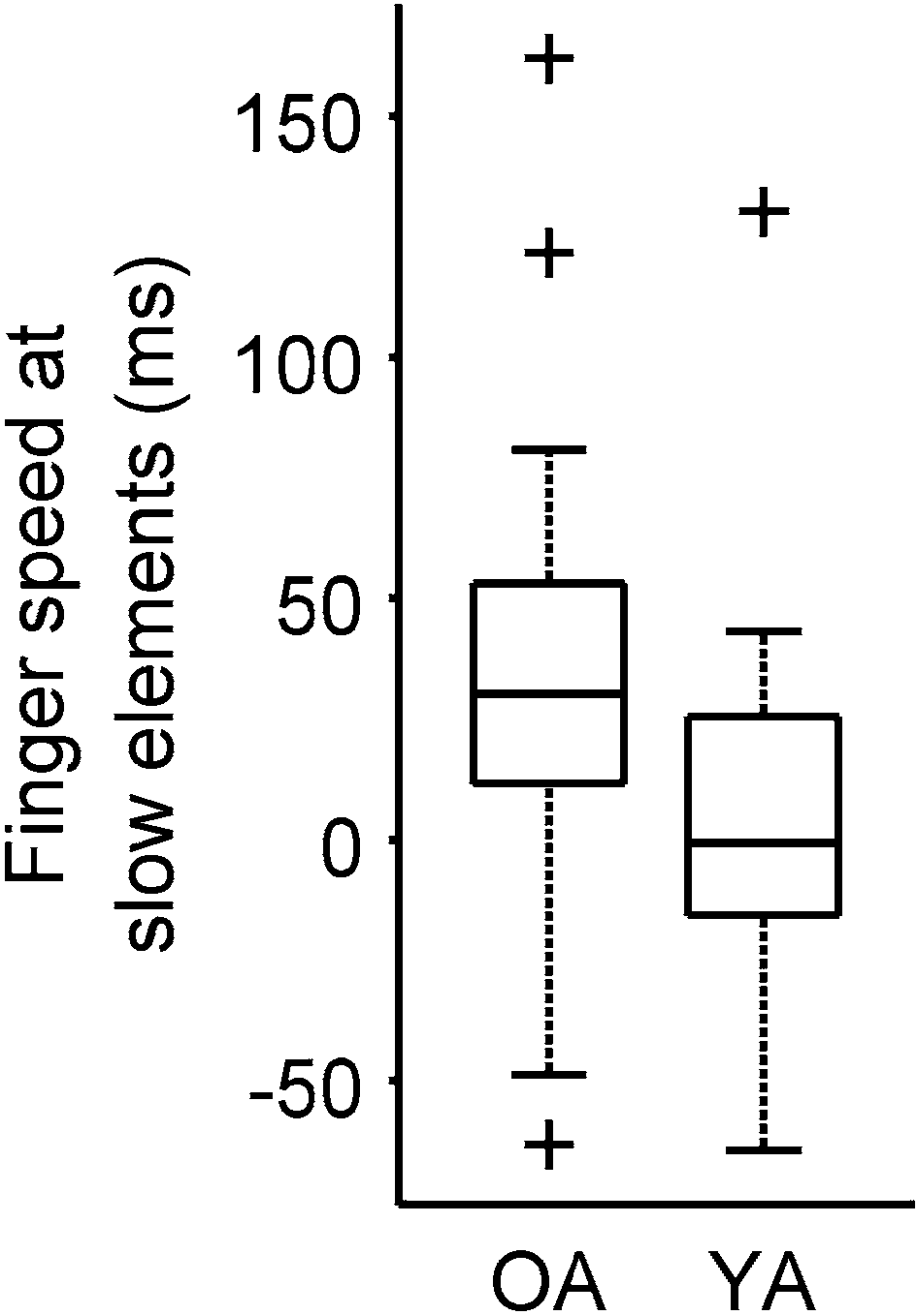
Finger speed at slow elements. The higher the value, the slower the fingers that were used at slow elements. *OA* older adults, *YA* young adults

Using the aforementioned method, we calculated the mean finger speed at the slow elements for all participants. We then tested the resulting distribution for older and young adults against zero. A two-tailed, one-sample *t* test shows that for older participants, the finger speed at slow elements was slower than the mean finger speed, *t*(17) = 2.6, *p* = 0.02 (see Fig. 5). For young participants, the finger speed at slow elements was not significantly different from the mean, *t*(17) = 0.7, *p* = 0.52. The difference between older and young participants was not significant either, *t*(34) = 1.7, *p* = 0.105.

### Awareness, visuospatial working memory capacity and processing speed

Both age groups showed a ceiling effect on explicit knowledge, with 9 out of 18 older and 12 out of 18 young participants reaching the maximum score. A Spearman’s rho correlation analysis shows that explicit knowledge and the familiar test-phase IED were correlated for older, *r*_s_ = 0.65, *p* = 0.004, but not for young participants. Regarding the VSWM task, most of the older participants mentioned that, although they understood the task well, they found the 100 ms presentation of the sample array (that was to be remembered and compared to an array presented 900 ms later) too short. Since the mean capacity for our older sample is 1.87 and thus higher than the mean capacity of 1.76 found previously a sample with a mean age of 71 (Bo et al., 2009), we decided to report the results regardless. As expected, young participants had a larger VSWM capacity than older participants (VSWM capacity 4.52 vs. 1.87), *t*(34) = 6.06, *p* < 0.001. Contrary to our hypothesis however, VSWM capacity did not correlate with the familiar test-phase IED for older, *r*(16) = 0.07, *p* = 0.775, or for young participants, *r*(16) = 0.0, *p* = 984. Young adults substituted more elements in the digit symbol substitution task than older participants (74 vs. 44 elements) and thus had a substantially higher processing speed, *t*(34) = 9.14, *p* < 0.001. However, the number of items substituted did not correlate with the familiar test-phase IED for older, *r*(16) = 0.39, *p* = 0.115, or for young adults, *r*(16) = 0.14, *p* = 0.582. Processing speed did correlate with execution rate in the random test condition for older, *r*(16) = − 0.51, *p* = 0.031, but not for young participants, *r*(16) = − 0.01, *p* = 0.959.

### Results summary

In line with our hypothesis, older adults continued to increase their IED during the second day, while their young counterparts had already reached a ceiling during the first day. The difference in IED between the age groups eventually diminished. In older adults, slow elements in familiar sequences were associated with a finger that was also slow during performance of the random sequences; such an effect was not found for the young. Older adults with more explicit knowledge showed a higher IED. The IED was not associated with VSWM or with processing speed in either age group.

## Discussion

Our primary goal was to test the hypothesis that extended practice would enable older adults to develop motor chunking behavior, which we measured using the initiation–execution difference (IED). Our hypothesis was supported, with the IED reaching almost the same level in the older as in the young adults. This indicates that older adults prepared learned sequences before movement onset to a similar extent as young adults, and thus showed similar chunking behavior. While the pattern of IED development was rather similar for both sequence lengths (see Fig. 4), the second day of practice led to a significantly increased IED in older adults for the 6- but not the 3-element sequence. Apparently, it took the older adults more time to develop chunking behavior for the longer sequence. For younger adults, the IED increased faster for the 6-element sequence than for the 3-element sequence. This may be due to accumulating activation in the associative mode which is used early in practice (Verwey & Abrahamse, 2012).

The IED was not equally robust in both age groups, especially during the first day of practice. It seems that the breaks and unrelated tasks between successive blocks of DSP practice negatively affected the IED for older but not for young adults (e.g., see block 6 in Fig. 4). The switch from the first to the second day also negatively affected the IED in the older sample. Previous research found reductions in older adults’ sleep-dependent consolidation for sequence performance in general (Gudberg, Wulff, & Johansen-Berg, 2015; Wilson, Baran, Pace-Schott, Ivry, & Spencer, 2012) and for motor chunking in particular (Bottary, Sonni, Wright, & Spencer, 2016). Our results suggest that for older adults performance on the next day can even be worse than on the previous day. Overall, the older adults in our study showed slower development of chunking for the 6-element sequence, but they did manage to develop chunking behavior after extended practice.

Our analysis of the effects of biomechanical variation between fingers on the occurrence of slow elements confirmed our expectations. For the first time, we show that for young adults slow elements in familiar sequences were not associated with finger speed in random sequences and, hence, the DPM’s interpretation that these elements are locations where motor chunks are concatenated need not be rejected (Abrahamse et al., 2013). In contrast, for older adults slow elements in the learned sequences were associated with fingers we identified as slow using data from the random sequence test condition. It is difficult to estimate to what extent cognitive and biomechanical factors contributed to the slowing of individual sequence elements in the older adults, but these findings suggest that potential indications for concatenation points in this group may in fact have been caused by biomechanical factors (like a stiff finger) rather than cognitive factors (like a concatenation point). As such, the occurrence of occasional slow responses in older adults alone is not sufficient evidence to support the use of motor chunks. An interesting question for further research that emerges from this finding is whether people in general, and older adults in particular, use the additional time introduced by a slow effector to perform additional cognitive processing.

The results presented here regarding chunking behavior and the effects of biomechanical variation provide relevant new insights, but also call for follow-up research to provide more detailed ideas regarding the analysis of sequence learning data. Many factors may affect RTs including, but not limited to, stiff fingers, handedness, wrist rotation, switching the hand used, and differences between fingers (e.g., the ring and little fingers have been found to be slower in piano studies, Aoki, Furuya, & Kinoshita, 2005). Previous research has proposed multiple analyses to analyze, sometimes rather specific, aspects of chunking. The methods used include *t* tests (Bo et al., 2009; Ruitenberg et al., 2014), k-means clustering (Song & Cohen 2014), dynamic network analyses (Wymbs, Bassett, Mucha, Porter, & Grafton, 2012), hidden Markov models (Acuna et al., 2014) and non-parametric rank-order algorithms (Alamia, Solopchuk, Olivier, & Zenon, 2016). An elaborate discussion of each of these methods is beyond the scope of the current work. There were several reasons why we choose for the IED as a measure of chunking. First, the IED provides an estimate of the strength, or extent, of chunking at different moments of practice. Second, it takes into account the potential confounding effects of fingers that are slow in general. Third, chunking structures differ per participant (Verwey & Eikelboom, 2003), something that our analysis took into account. Finally, using this method allows us to, indirectly, compare outcomes of our study to previous DSP chunking studies in older adults (Verwey, 2010; Verwey et al., 2011) A downside of our t test based method that we could not overcome is that of the 6 elements, only RT 3, 4 and 5 can be a slow element. The result is that when a finger is very slow in the random sequences, but not used at RT 3, 4 or 5, it is not included in our findings regarding finger speed at slow elements. Note that while this downside makes our approach less sensitive, the present finding of a relationship between slow elements and general finger speed only underlines robustness of this finding.

Next to our primary goals, we explored how chunking behavior was related to explicit knowledge, VSWM, and processing speed. Explicit sequence knowledge was correlated with the IED for older adults in keeping with previous findings (Verwey, 2010), suggesting that when older adults are executing keying sequences this may not solely rely on pure motor representations. This is in line with the recently proposed cognitive framework for sequential motor behavior (C-SMB, Verwey et al., 2015), built on the foundations of the DPM, which postulates that the representations underlying motor skill and motor chunks may be mixed. That is, verbal and/or visuospatial central-symbolic representations may underlie skilled sequence performance too. A post hoc interpretation may then be that a mixture of motor and cognitive sequence knowledge drives the chunking mode displayed by older adults. In line with the previous suggestions of enhanced analyses, future research could focus on teasing apart the specific representations underlying chunking behavior.

The expected relationship between VSWM and chunking behavior (in terms of IED) was not observed in either age group. For the older participants, this may be related to the difficulties they reported during the VSWM task. It may also be that the extent of preparation and chunking, as quantified with the IED, is independent of the actual VSWM capacity. Remember that the IED measure also reflects the time uncertainty that is associated with the first response of a discrete sequence. Our results suggest that processing speed does not play a strong role in chunking behavior. This fits the idea that older adults had still been relying more on the motor processor than on the cognitive processor (Abrahamse et al., 2013). That processing speed in older adults is indeed associated with the cognitive processor was indicated by the present finding that processing speed did correlate with random sequence performance.

In summary, we found that age differences in motor chunking behavior, as measured by the difference between preparation and execution of the sequence (i.e., IED), diminish with extended practice. Unlike young adults, older adults appeared to show an association between slow elements in the sequences and fingers that were slow in the random sequences. This finding shows that future research should take into account the possibility that in older adults a slow sequence element may be caused by a slow, perhaps stiff, finger instead of by the start of a next motor chunk.

## Footnotes

1 We intentionally chose a terminology that is not related to any theoretical cognitive concept and refrain from using terms like ‘chunk point’ or ‘concatenation point’ since our goal is to establish whether purely biomechanical factors, unrelated to cognitive processes play a role in the occurrence of slow elements.

2 RT1 is not a suitable neighbor because it is usually slower because of time uncertainty in the presentation of the first stimulus of the sequence.

3 In the 6-element sequences, each of the six fingers is used once. This means that when any of a participant’s fingers is consistently slow, this will show up as a slow response at one point in the sequence.
